# A survey of thrombosis experts evaluating practices and opinions regarding venous thromboprophylaxis in patients post major abdominal surgery

**DOI:** 10.1186/s12959-016-0126-9

**Published:** 2017-01-13

**Authors:** Bader Al Rawahi, Grégoire Le Gal, Rebecca Auer, Marc Carrier

**Affiliations:** 1 Department of Hematology, Sultan Qaboos University, Sultan Qaboos University Hospital, P.O.Box 38, Al-Khoud, 123 Sultanate of Oman; 2 Department of Medicine, The Ottawa Hospital Research Institute, University of Ottawa, Ottawa, Canada; 3 Department of Surgery, The Ottawa Hospital Research Institute, University of Ottawa, Ottawa, Canada

## Abstract

**Background:**

Patients undergoing major abdominal surgery are at high risk for developing venous thromboembolism in the post-operative period. Current evidence-based guidelines recommend routine pharmacological venous thromboembolism prophylaxis in patient at moderate to high risk post major abdominal surgery. However, the type of agent, dose and duration of thromboprophylaxis remain unclear. We sought to survey current clinical practice and assess for potential clinical equipoise regarding pharmacological thromboprophylaxis post major abdominal surgery.

**Methods:**

An electronic survey targeting thrombosis expert members of Thrombosis Canada was conducted.

**Results:**

The total response rate was 52.3% (45/86). All thrombosis experts recommended pharmacological thromboprophylaxis for high risk patients post major abdominal surgery. Over 68% of the thrombosis experts recommended thromboprophylaxis during hospitalization only. The majority of the participants recommended using LMWH (85.9%) over UFH (10.1%). Approximately a third of the surveyed thrombosis experts estimated the incidence of overall VTE at 7 to 10 days post-operatively in patients who do not receive thromboprophylaxis post major abdominal surgery to be between 4 and 6%. A total of 55.3% of the thrombosis experts estimated the incidence of PE to be between 0.5 and 1.0% for the same patient population. The risk of major bleeding episode was estimated to be between 0.5 and 1% in patients receiving 7 to 10 days of pharmacological thromboprophylaxis in the post-operative period by a majority of the thrombosis experts (68.4%). However, approximately 80% of thrombosis experts believed that there is still some clinical equipoise around the use of thromboprophylaxis post discharge (up to 7 to 10 days) in high risk adult patients post major abdominal surgery.

**Conclusions:**

Thrombosis experts recommend LMWH prophylaxis post major abdominal surgery. There is still, however, significant clinical equipoise regarding the duration of thromboprophylaxis (hospitalization only vs. total to 7–10 days). The result of the survey might not be generalizable to non-academic centers and to other countries.

**Electronic supplementary material:**

The online version of this article (doi:10.1186/s12959-016-0126-9) contains supplementary material, which is available to authorized users.

## Background

Venous thromboembolism (VTE) is a condition associated with an increased morbidity and mortality among hospitalized medical and post-surgical patients. The most common presentations of venous thromboembolism are deep vein thrombosis (DVT) of the lower extremity and pulmonary embolism (PE) [1]. Patients undergoing major abdominal surgery (include any abdominal surgery that is laparoscopic or open, performed under general anaesthesia and lasted for at least 30 min) are at risk of developing a VTE complication in the post-operative period. Their VTE risk depends on both patient-specific and procedure specific factors [2]. Old age, previous VTE, cancer, obesity and prolonged immobilization post-surgery are examples of high-risk patient-specific factors. Examples of high-risk procedures include open abdominal and pelvic surgeries, abdominal-pelvic cancer surgery and bariatric surgery. Based on those risk factors, the estimated baseline risk for VTE post major abdominal surgery in patients with high risk factors for VTE is approximately 6% [2].

The American College of Chest Physician (ACCP) Evidence-based consensus guidelines published in 2012 [2] recommend that patients undergoing non-orthopedic surgery at moderate or high risk for VTE (general, abdominal-pelvic or thoracic surgeries) receive routine pharmacological thromboprophylaxis (Low molecular weight heparin (LMWH), unfractionated heparin (UFH) or fondaparinux). Although the efficacy and safety of pharmacological thromboprophylaxis agents have been proven, which agent to use (e.g. UFH vs. LMWH vs. fondaparinux) and at which dose (e.g. UFH 5,000 IU every 8 or 12 h) remains debatable. Furthermore, the duration of pharmacological thromboprophylaxis (i.e. in-hospital only vs. 7 to 10 days including an outpatient prescription) is unclear. We sought to establish the current clinical practice of Canadian thrombosis experts, assess for potential clinical equipoise regarding pharmacological thromboprophylaxis in this patient population and evaluate the potential participation in a future randomized clinical trial.

## Methods

### Population

The survey targeted thrombosis expert members of Thrombosis Canada. Thrombosis Canada is an established group of expert Canadian clinicians dedicated to advancing education and research in the prevention and treatment of thrombo-vascular disease [3]. Thrombosis experts of Thrombosis Canada are defined as Canadian clinicians who have made many significant contributions to the body of knowledge in vascular medicine and disseminated that knowledge through peer reviewed journal publications and books as well authoring national and international clinical practice guidelines. This expert clinician group has the necessary expertise and experience to provide meaningful opinions on the planning of a potential future randomized clinical trial (RCT).

Survey Monkey [4] online software was used to create and distribute the survey. Each survey participant received an email with hyperlink to the survey. A reminder email with a link to the survey was sent weekly for 2 weeks. Our target response rate was 35% based on previously published response rate of physician specialist to web-based survey [5]. The survey included a short introduction to the survey, its goals/objectives and the reason why the participant was chosen to participate. This was followed by series of categorical questions (a total of 14) with 4–5 answers, based on a short clinical vignette (Please see Additional file 1: Appendix 1 on-line). The first few questions were about the participant’s current clinical practice. The following questions were related to two different clinical scenarios. We surveyed participants on their opinions on the efficacy and safety of pharmacological thromboprophylaxis and assessed if equipoise still exist around its post major abdominal surgery. Finally, we asked the participants if they would consider including their patients in a RCT, and if yes, to what intervention, dose and duration. Participation in the survey was voluntary and all data were kept anonymous and confidential. Filling out the online survey was viewed as an implied consent. All response answers were saved in the Survey Monkey online program, which was later spread into Microsoft excel program in the form of pooled data for analysis. Data was analyzed after 2 months of sending the survey.

Descriptive statistics (percentages) were used to analyze and summarize the result of the survey. Simple percentage compression were made between relevant demographic subgroups. Analyses were conducted using Survey Monkey online program.

## Results

The initial response rate was 34.9% (30/86) and the total response rate was 52.3% (45/86), of which only 37 completed the whole survey (43%).

The majority of the participants were hematologists (40.5%) followed by internists (29.7%). Most participants were males (67.6%) and middle age adults (age 46–55 (35.1%)) and the majority were in clinical practice for more than 10 years (56.8%). Most responders were from the province of Ontario (59.5%) and the majority (83.8%) of the thrombosis experts practiced in an academic center.

All the thrombosis experts recommended the use of thromboprophylaxis post major abdominal surgery. Approximately 70% percent (68.9%) recommended using thromboprophylaxis during hospitalization only. The others recommended extending thromboprophylaxis for 7–10 days or for a total of 28 days post major abdominal surgery (26.7 and 4.4% respectively).

The majority of the thrombosis experts (85.9%) recommended LMWH over UFH. Dalteparin 5,000 units daily or enoxaparin 40 mg daily were the most frequently recommended regimens (33.3 and 24.2%, respectively).

Approximately a third of the thrombosis experts estimated the incidence of overall VTE (symptomatic and asymptomatic) at 7 to 10 days post-operatively in patients who do not receive thromboprophylaxis post major abdominal surgery to be between 4 and 6% whereas 55.3% estimated the incidence of PE to be between 0.5 and 1.0% in this patient population. The risk of major bleeding episode was estimated to be between 0.5 and 1% in patients receiving 7 to 10 days of pharmacological thromboprophylaxis in the post-operative period by a majority of the participants (68.4%). Finally, a majority of thrombosis experts (57.9%) believe that the benefits of using pharmacological thromboprophylaxis for 7 to 10 days in high-risk patients outweigh the risk of bleeding in adult patients post major abdominal surgery in most cases. However, approximately 80% thrombosis experts believe that there is still some clinical equipoise especially around the use of thromboprophylaxis post discharge (up to 7 to 10 days) in high risk adult patients post major abdominal surgery. Thus, it is not surprising that they would consider allowing their patients to participate in a RCT assessing the use of thromboprophylaxis in adult patients post major abdominal surgery comparing different duration (e.g. during hospitalization only vs. 10 days) of thromboprophylaxis (89.5%).

## Discussion

This survey of Canadian thrombosis experts shows that there is an agreement in the use of pharmacological thromboprophylaxis post major abdominal surgery. It also shows that majority of the experts would use thromboprophylaxis during hospitalization only. It also confirms that there is clinical equipoise and uncertainty around the use of thromboprophylaxis post discharge (up to 7 to 10 days) in high-risk adult patients post major abdominal surgery and that a clinical trial is desirable.

A majority of the clinicians selected LMWH as their preferred pharmacological thromboprophylactic agent. This is not surprising given that LMWH have a better safety profile compared to UFH. Unfractionated heparin requires subcutaneous self-injections twice or three times daily making them less convenient especially for extended post discharge thromboprophylaxis. In addition, UFH is associated with 2.6% risk of heparin induced thrombocytopenia (HIT), a rare but potentially serious adverse reaction causing low platelets with paradoxical thrombosis and tissue necrosis [2]. LMWH is less likely to cause HIT (0.2% compared to 2.6% with UFH) [2]. Although it is also given subcutaneously, it is usually given less frequently, usually once daily making it more appealing than UFH for extended post discharge thromboprophylaxis.

The majority of participants estimated the incidence of PE between (0.5–1.0%) closely to the pooled estimates previously reported (0.5%) [6]. However, they under estimated the incidence of overall VTE at (4–6%) and major bleed (0.5–1.0%) post major abdominal surgery (14.5 and 2.8%, respectively) [6].

It was not surprising that there is an agreement regarding clinical equipoise around the use of thromboprophylaxis post discharge (up to 7 to 10 days) in high–risk adult patients post major abdominal surgery. Although most of clinical trials evaluated different pharmacological thromboprophylaxis for a fixed duration of 7 to 10 days, the surgical techniques, post-operative management and length of stay have changed significantly over recent years and more contemporary data is desperately needed. Furthermore, there is a lack of clinical trials that directly compared two different durations of thromboprophylaxis (in-hospital only vs. 7 to 10 days). Thus, the majoring of the experts would consider participating in a clinical trial comparing two different durations of thromboprophylaxis.

It is important to acknowledge the limitations of our cross sectional study. The survey was limited to Canadian experts, mostly from academic centers, and therefore may not reflect the opinion of other international experts or clinicians and surgeons in community hospitals. Similarly, the survey was not validated and tested in other populations. It would have been ideal to also capture the opinions of the general surgeons. We piloted the survey in a subgroup of members of the Canadian Association of General Surgeons [7] . However, it was felt that the questions of VTE and major bleeding complication rates were beyond the scope of their practice and be better defined by a group of Thrombosis Medicine. Therefore, we surveyed experts in the field to provide the most significant and applicable opinion in the topic. Similarly, the membership of Thrombosis Canada is of relatively small size and this might have resulted in potential selection bias. Nonetheless, they remained the most important clinical experts to survey. In addition, although the overall response rate can be still considered low, it exceeded our targeted response rate.

## Conclusion

There is an agreement among thrombosis experts in using LMWH for thromboprophylaxis post major abdominal surgery. There is still equipoise around the use of pharmacological thromboprophylaxis for 7–10 days post-operatively including post discharge prescription. There sms to be underestimation of major bleeding events post major surgery in patients receiving pharmacological thromboprophylaxis. There is a need for a RCT comparing the use of pharmacological thromboprophylaxis in hospital only compared to duration of 7–10 days (including post discharge prescription) post major abdominal surgery.

## Additional file

Additional file 1: Survey Questionnaires. (DOCX 17 kb)
