# Supplementary material for: A survey of thrombosis experts evaluating practices and opinions regarding venous thromboprophylaxis in patients post major abdominal surgery
Source: Thromb J. 2017 Jan 13;15:2. doi: 10.1186/s12959-016-0126-9 (PMC5237241; doi:10.1186/s12959-016-0126-9)
Supplement: Additional file 1: — Survey Questionnaires. (DOCX 17 kb) [file 12959_2016_126_MOESM1_ESM.docx]

# Appendix 1: Survey Questionnaire

###

***A Cross Sectional Survey: A Survey Of Thrombosis Experts Evaluating Practices And Opinions Regarding Venous Thromboprophylaxis In Patients Post Major Abdominal Surgery+***

Major abdominal surgery: include any abdominal surgery that is laparoscopic or open, performed under general anaesthesia and lasted for at least 30 minutes.

1. Questions 1 and 2 are about your clinical practice

A 49 year-old male (BMI = 30 kg/m^2^) undergoing laparoscopic colon resection (> 45 minutes) for ulcerative colitis that is refractory to medical therapy. He is to be hospitalized for 1-2 days after surgery (Caprini score of 5).

1. **Currently in your practice would you recommend the use of pharmacological thromboprophylaxis for this patient?**
   - Yes. While admitted to hospital only
   - Yes. For a total of 7 to 10 days
   - Yes. For a total of 28 days
   - I would not recommend any pharmacological thromboprophylaxis
   - Other? Please specify: ------.
2. **Which types/dose/schedules of pharmacological thromboprophylaxis do you recommend (weight = 70 kg and normal renal function)? (please mark all that apply)**
   - Unfractionated Heparin (UFH) 5,000 units, subcutaneous (SC), twice a day.
   - UFH 5,000 units, SC, three times a day.
   - Enoxaparin 30mg, SC, twice a day.
   - Enoxaparin 40mg, SC, daily.
   - Dalteparin 5,000 units, SC, daily.
   - Tinzaparin 4,500 units or 75 units/kg, SC, daily.
   - Fondaparinux 2.5mg, SC, daily.
   - Aspirin 75-325 mg, PO, daily.
   - Never
   - Other? Please specify: ------.

B. Questions 3-11 are about your opinion and use of clinical practice guidelines for thromboprophylaxis in adult patients post major abdominal surgery:

A 49 year-old male (BMI = 30 kg/m^2^) undergoing laparoscopic colon resection (> 45 minutes) for ulcerative colitis that is refractory to medical therapy. He is to be hospitalized for 1-2 days after surgery (Caprini score of 5).

1. **In your opinion, in the case above, what is the probability (incidence) of overall venous thromboembolism (VTE) (symptomatic and asymptomatic VTE (i.e. including screening ultrasound or venography) at 7 to 10 days post-operatively if this patient does not receive thromboprophylaxis?**
   - 1-3%.
   - 4-6%.
   - 7-9%.
   - 10-12%.
   - >=13%.
2. **In your opinion, in the case above, what do you think is the probability (without thromboprophylaxis) of symptomatic pulmonary embolism at 7 to 10 days post-op?**
   - 0.5-1%.
   - 2-3%.
   - 4-5%.
   - 6-7%.
   - >=8%.
3. **In your opinion, in the case above, what is the incidence of a major bleeding episode* if the patient receiving 7 to 10 days of pharmacological thromboprophylaxis in the post-operative period?**

*****Major bleeding: Fatal bleeding, and/or symptomatic bleeding in a critical area or organ, such as intracranial, intra-spinal, intraocular, retroperitoneal, intra-articular or pericardial, or intramuscular with compartment syndrome, and/or bleeding causing a fall in hemoglobin level of 20 g/l or more, or leading to transfusion of two or more units of whole blood or red cells and/or bleeding required reoperation to stop the bleeding.

- - 0.5-1%.
  - 2-3%.
  - 4-5%.
  - 6-7%.
  - >=8%.

1. **We performed a systematic review of the literature on the efficacy and safety of pharmacological thromboprophylaxis in patients undergoing major abdominal surgery. A large majority of clinical studies assessed pharmacological thromboprophylaxis for a total of 7 to 10 days. There are no clinical trials comparing a course of pharmacological thromboprophylaxis during the hospitalization only to a total duration of 7 to 10 days in this patient population. Therefore, some clinical practice guidelines suggest to consider a minimum 7 to 10 days of pharmacological thromboprophylaxis (including post-discharge prescription if applicable) for high risk patients undergoing major abdominal surgery. In your opinion, do you think that the benefits of using pharmacological thromboprophylaxis for 7 to 10 days in high risk patients outweigh the risk of bleeding in adult patients post major abdominal surgery?**
   - Always.
   - Most of the time
   - Sometimes.
   - Never.
   - Other? Please specify: ------.
2. **Do you believe that there is clinical equipoise around the use of thromboprophylaxis post discharge (up to 7 to 10 days) in high risk adult patients post major abdominal surgery??**
   - Yes.
   - No.
3. **Would you consider allowing your patients to participate in a randomized trial assessing the use of thromboprophylaxis in adult patients post major abdominal surgery (please choose only one answer)?**
   - Yes, comparing thromboprophylaxis to placebo.
   - Yes, comparing different duration (e.g. during hospitalization only vs. 10 days) of thromboprophylaxis or agents.
   - No, I would not enroll patients post major abdominal surgery in a clinical trial.

C Questions 9-14 are about you and your clinical practice:

1. **You are a:**
   - Hematologist**.**
   - Internist**.**
   - Family Doctor
   - Other? Please specify_______________.
2. **You are a:**
   - Male**.**
   - Female.
3. **Your age is:**
   - 25-35**.**
   - 36-45**.**
   - 46-55**.**
   - 56-65**.**
   - over 65
4. **Your have been in independent practice for ________years.**
5. **You practice >50% of your time in the province of:**
   - NF.
   - NS.
   - NB.
   - PE**.**
   - QC.
   - ON**.**
   - MB.
   - SK.
   - AB.
   - BC**.**
   - YT/NT /Nunavut
6. **You practice >50% of your time at:**
   - A non-academic (community) hospital.
   - An academic (teaching) hospital.
   - Private Practice Office.
   - Other? Please specify______________.
